# Actinorhizal Alder Phytostabilization Alters Microbial Community Dynamics in Gold Mine Waste Rock from Northern Quebec: A Greenhouse Study

**DOI:** 10.1371/journal.pone.0150181

**Published:** 2016-02-29

**Authors:** Katrina L. Callender, Sébastien Roy, Damase P. Khasa, Lyle G. Whyte, Charles W. Greer

**Affiliations:** 1 Department of Natural Resource Sciences, McGill University, Ste-Anne-de-Bellevue, Quebec, Canada; 2 Energy, Mining and the Environment, National Research Council, Montreal, Quebec, Canada; 3 Department of Biology, Université de Sherbrooke, Quebec, Canada; 4 Université Laval, Québec, Canada; University of Oklahoma, UNITED STATES

## Abstract

Phytotechnologies are rapidly replacing conventional *ex-situ* remediation techniques as they have the added benefit of restoring aesthetic value, important in the reclamation of mine sites. Alders are pioneer species that can tolerate and proliferate in nutrient-poor, contaminated environments, largely due to symbiotic root associations with the N_2_-fixing bacteria, *Frankia* and ectomycorrhizal (ECM) fungi. In this study, we investigated the growth of two *Frankia*-inoculated (actinorhizal) alder species, *A*. *crispa* and *A*. *glutinosa*, in gold mine waste rock from northern Quebec. Alder species had similar survival rates and positively impacted soil quality and physico-chemical properties in similar ways, restoring soil pH to neutrality and reducing extractable metals up to two-fold, while not hyperaccumulating them into above-ground plant biomass. *A*. *glutinosa* outperformed *A*. *crispa* in terms of growth, as estimated by the seedling volume index (SVI), and root length. Pyrosequencing of the bacterial 16S rRNA gene for bacteria and the ribosomal internal transcribed spacer (ITS) region for fungi provided a comprehensive, direct characterization of microbial communities in gold mine waste rock and fine tailings. Plant- and treatment-specific shifts in soil microbial community compositions were observed in planted mine residues. Shannon diversity and the abundance of microbes involved in key ecosystem processes such as contaminant degradation (*Sphingomonas*, *Sphingobium* and *Pseudomonas*), metal sequestration (*Brevundimonas* and *Caulobacter*) and N_2_-fixation (*Azotobacter*, *Mesorhizobium*, *Rhizobium* and *Pseudomonas*) increased over time, i.e., as plants established in mine waste rock. Acetate mineralization and most probable number (MPN) assays showed that revegetation positively stimulated both bulk and rhizosphere communities, increasing microbial density (biomass increase of 2 orders of magnitude) and mineralization (five-fold). Genomic techniques proved useful in investigating tripartite (plant-bacteria-fungi) interactions during phytostabilization, contributing to our knowledge in this field of study.

## Introduction

Surface mining operations, such as those at Val d’Or, Quebec, comprise 90% of the world’s mineral output. Open-pit mines generate up to ten times more waste than underground mines due to their sheer size, the nature of the ore deposits and the extraction processes required. Surface mining involves the excavation and retrieval of metal-bearing ore on or near the Earth’s surface via blasting using high explosives. Cyanidation and zinc precipitation are then utilized for gold extraction and refining purposes [1]. An estimated 30 tons of mine waste are generated for every ounce of gold produced and the average lifetime of a metal mine is under 20 years [2]. As a result of the sheer volume of material that can be extracted and processed per day, surface mines drastically alter land characteristics, topography and composition.

The Canadian mining industry includes over 800 metal and mineral mines and produces up to 650 million tons of waste per year in the form of tailings, waste rock, overburden and loose soil piles. This waste is often metal-contaminated and in addition to being an eyesore, has the ability to contaminate land and water resources of adjacent communities.

Phytoremediation involves the use of plants and their associated microbes to extract, degrade, contain (stabilize) or remove contaminants in situ. Phytotechnologies such as phytostablization are rapidly gaining popularity as an aesthetic, cost-effective alternative to conventional mine reclamation practices such as capping, excavation and chemical stabilization. They require no or minimal nutrient input and can treat multiple contaminants and residual contamination from both metallic and organic compounds. This is because plants stimulate the growth and maintenance of soil microbial communities leading to continuous soil decontamination, improved soil quality and restoration of ecosystem function. As an additional benefit, plants form a biological cap, preventing the spread of contaminants via aeolian and water erosion [3].

Gold mine waste rock at Val d’Or is moderately metal-contaminated, slightly alkaline (pH 8.6) and has poor soil characteristics, including low water retention and low fertility, as it is devoid of organic matter and macronutrients. Although the nature and composition of these mine residues makes them challenging substrates for plant growth, revegetation is crucial to improve soil structure, quality and fertility. The re-establishment of a natural forest reduces soil erosion while increasing soil water retention and organic matter content, which contributes to soil stability [4].

Actinorhizal plants such as alders, pioneer trees and shrubs in many disturbed lands, can tolerate harsh environments and promote secondary and successive plant species through the addition of soil nutrients, especially nitrogen, leading to restored ecosystem health and function [5]. This is due to their ready establishment, rapid growth, abundant litter production and ability to host symbiotic root associations with N_2_-fixing bacteria such as *Frankia* in root nodules as well as ectomycorrhizal (ECM) fungi on root tips [6]. Given that they do not hyperaccumulate or translocate trace and heavy metals [7], they are excellent phytostabilizing plant species and have been successfully used for strip-mine reclamation in a wide variety of environments ranging from oil sands process affected material (OSPM) to heavy metal-contaminated mine spoils. Inoculating alders with functional (effective) *Frankia* strains reduces phytotoxicity and subsequently enhances plant biomass production in heavy metal-contaminated soil [6]. Actinorhizal plants often enter symbiosis spontaneously with ectomycorrhizal (ECM) and arbuscular fungi [8] These tripartite interactions, i.e., symbiotic associations of alders with nitrogen-fixing bacteria and mycorrhizal fungi, have been shown to act synergistically to improve plant (including actinorhizal species) performance when grown on heavy metal-contaminated mine tailings [9].

The successful application of *Alnus rugosa* in the revegetation of the Gays River lead-zinc mine tailings in Nova Scotia, Canada was first demonstrated in 1988 [10]. In more recent studies, alders have demonstrated an impressive ability to restore disturbed lands ranging from metal mine overburden, waste rock and tailings to oil sands tailings, while positively impacting soil fertility and increasing soil total nitrogen and carbon [11–23] *Frankia* inoculation prior to transplantation into mine residues has been shown to be beneficial to the growth and establishment of alders in mine residues [9, 16; 24–25]. Actinorhizal alders were also successfully grown in tailings sand and composite tailings, where they positively impacted the diversity and activity of the indigenous soil microbial populations [13].

Soil microbes play key roles in important ecosystem processes such as nutrient (nitrogen, phosphorus, sulfur) cycling, carbon cycling and soil formation [26–31]. They also regulate plant productivity, especially in nutrient poor ecosystems where plant root symbionts are responsible for the acquisition of limiting nutrients. N_2_-fixing bacteria and ECM fungi, for example, account for approximately 80% of all nitrogen, and up to 75% of phosphorus, acquired by plants (in temperate and boreal forests) annually [32]. The sensitivity, rapid response, and integrative character of biological indicators of soil health, such as the size, activity, and diversity of soil microbial communities [33, 34], make them invaluable tools for assessing the efficiency and success of metal phytostabilization [35].

Approximately 60% of the Earth’s biomass is microbial, the vast majority of which have not yet been cultured. Metagenomics, the study of genetic material recovered directly from environmental samples (metagenomes), uses DNA sequencing-based techniques, bypassing the need for isolation and lab cultivation of individual species. Having the ability to directly access genomes of hard-to-study organisms and complex environments, metagenomics and next-generation sequencing (NGS) technologies have considerably advanced our understanding of the genomic diversity within natural environments, microbial species interrelationships, environmental-niche adaptations and the dynamics of whole microbial communities, including the environmental factors that shape them, such as soil pH, texture and nutrient status [36]. NGS technologies provide unparalleled insight into community structure, phylogeny and taxonomy relative to capillary (Sanger) sequencing and non-sequence-based molecular methods. The resolution of the community composition with amplicon pyrosequencing is potentially several orders of magnitude larger than clone library sequencing, and can be achieved at a significantly lower cost [37]. The nine different variable 16S rRNA gene regions (V1-V9) are flanked by conserved stretches in most bacteria [38], and they can be used as targets for PCR primers with near-universal bacterial specificity [39, 40]. While 18S rRNA genes are highly conserved in fungi, internal transcribed spacer (ITS) regions contain both highly variable and highly conserved base sequences [41]. The ITS region is the most widely sequenced DNA region in fungi and has been recommended as the universal fungal barcode sequence. PCR amplification and pyrosequencing of 16S rRNA genes in bacterial or internal transcribed spacer (ITS) regions is a commonly used method for studying phylogeny and taxonomy and community structure, particularly in complex samples or uncultivable microorganisms. In fact, the majority of bacterial phyla are known only from 16S rRNA surveys and have no cultured representatives [42–45]

While microbial communities in acid mine drainage (AMD) have been extensively studied due to the extreme pH conditions (pH < 3.0) and high concentrations of heavy metals, influences on microbial community structure and function are not yet fully understood [46–49]: both neutral and negative impacts on soil microbial activity and diversity have been previously observed [50, 51]. Less is known about microbial communities in neutral to alkaline (pH 5.5–8.5) mine environments including waste rock piles, tailings and neutral mine drainage (NMD) as these sites have not been widely studied [50, 52, 53].

In this study, we successfully exploited the natural symbiotic association of alders with *Frankia* and ECM fungi to revegetate waste rock from an active gold mine. Specifically, our objectives were to; i) evaluate the growth and performance of two alders, green or Mountain alder (*Alnus crispa*) and common or European alder (*Alnus glutinosa*); ii) assess the impacts on soil quality and metal fate; and iii) characterize changes in soil microbial community diversity and activity.

## Materials & Methods

### Experimental design

Two actinorhizal alder species typically found in boreal forests (taiga), *Alnus viridis ssp*. *crispa* (green or mountain alder) and *Alnus glutinosa* (common or European alder) native to and naturalized in, respectively, Canada were used in this experiment. These alders are important in primary and secondary taiga succession, and in many cases, comprise part of the natural vegetation encroaching mines as well as other degraded and disturbed ecosystems [54] In fact, *A*. *glutinosa* has been previously shown to perform well on contaminated sites in Western Canada [16], Seedlings of *Alnus glutinosa*, a tree species, and *Alnus viridis* ssp. *crispa*, a shrub species, were started in a greenhouse in September 2012. Alder seeds (obtained from the National Tree Seed Centre: *Alnus glutinosa* #8180890.0, Turkey, and *Alnus viridis* ssp. *crispa* #8360546.3, Obed Summit, Alberta) were cold stratified on Whatman No. 1 filter paper in petri dishes at 4°C for 48 hrs and then seeded, 10 seeds/pot (*A*. *crispa* and *A*. *glutinosa* seedlings have low germination rates; ≤ 34.5%), directly into 175 cm^3^ Rootrainer™cells containing a 3:1 (v/v) peat moss-vermiculite mixture and allowed to germinate (within 7–10 days) under natural light conditions in a greenhouse, watering as needed, typically every other day. To mimic normal nursery conditions, the greenhouse substrate was not sterilized and starting at two weeks post germination, seedlings were fertilized weekly with 0.1X Hoagland’s solution [55] containing 25 ppm N (NaNO_3_ added as the N source). Seedlings were thinned to one seedling per root trainer cell at 5 weeks post germination. At the 4-leaf stage, approximately 7 weeks post germination, seedlings were inoculated with *Frankia* sp. strain *AvcI1* diluted with NaCl (0.85%) to a target of 2 μl packed cell volume (pcv) per ml [56, 57]. The packed cell volume or pcv is a unit of measurement for *Frankia* describing the volume occupied by a cell pellet after centrifugation, since this bacterium forms thick aggregated biomass that is otherwise very difficult to quantify. Five (5) ml of *Frankia* inoculum was dispensed per plant for a total of 10 μl pcv/plant. Three months after germination, when the roots were well developed, *A*. *crispa* and *A*. *glutinosa* seedlings were transplanted to 1 litre pots containing a 3:1 (v/v) mixture of waste rock: fine tailings (moderately contaminated mine residues obtained from Val d’Or, QC) with or without the addition of 200 ml of maple woodchips as a soil amendment for a total of 4 treatments (plant species x amendment: *A*. *crispa* and *A*. *glutinosa* in unamended mine residues, Ac-NT and Ag-NT, respectively; and *A*. *crispa* and *A*. *glutinosa* in woodchip-amended mine residues, Ac-WC and Ag-WC, respectively) with 10 replicates (plants) each. No fertilization was done after transplanting into mine residues. Pots were randomly placed in the greenhouse and rotated weekly taking into account a potential light gradient, with each treatment kept separately. Alder height and root collar diameter were measured weekly in order to calculate the seedling volume index (SVI), an estimate of plant biomass defined as the square of the root collar diameter multiplied by the shoot height.

After six (6) months of growth, plants and soil (rhizosphere and bulk soil) were harvested for analysis.

### Sample collection and analysis

At the end of the six-month experiment, all surviving alders and three (3) unplanted, unamended controls were harvested for analysis. Soil was collected from controls first to reduce the risk of contamination. For each treatment, rhizosphere and bulk soil samples from five (5) randomly selected alders were used for pyrosequencing analysis, while composite soil samples were used for microbial enumeration and mineralization assays.

To sample alders the aerial portion of each plant was cut at the crown, approximately one (1) cm above the soil level. Roots were then removed from pots, shaken gently for 30 sec to remove excess soil, and then shaken vigorously to recover bulk soil.

For rhizosphere soil recovery, roots were shaken at 115 rpm for 90 min in sterile water (2 to 4 volumes the fresh weight to ensure roots were completely immersed). The resulting soil slurry was centrifuged at 12,400 x g for 10 min. The rhizosphere was the remaining soil pellet after the supernatant was discarded. Bulk and rhizosphere soils not immediately used were stored at –80°C. Ten grams dry weight (obtained by oven-drying overnight at 105°C) of soil per rhizosphere and bulk soil sample were sent to Maxxam Analytics (Montreal, QC, Canada) for chemical analysis, including total extractable metal concentrations.

After detaching the upper parts of alders from the roots at the crown, leaves and stems were separated, dried for 24h at 105°C, ground, combined and also sent to Maxxam Analytics for nutritional and metal analysis via ICP-MS.

Root nodules were aseptically separated from roots and both were surface sterilized using a modified protocol [58]. Roots were rinsed twice with sterile Milli-Q water and then subjected to a sterilization regime consisting of manually shaking roots for 1 min in 100% EtOH, 1 min in 2.5% bleach solution (NaClO) and agitating at 115 rpm for 10 min in fresh 2.5% bleach solution followed by a quick rinse with 100% ethanol. Roots were then rinsed four times with sterile Milli-Q water and a 1 ml aliquot of the final rinse was checked for sterility via PCR.

Root nodules were kept at 4°C and processed in the same way within 1–2 days. Surface sterilized roots were stored at –80°C.

### Microbial Enumeration Tests

Bulk and rhizosphere soil microbial biomass, specifically total heterotrophic bacteria (THB), were enumerated using a modified most probable number (MPN) technique [59]. Viable bacterial counts were determined in 96-well plates using YTS_250_ medium (250 mg each of yeast extract, bacto-tryptone and starch per litre of water). Plates were wrapped in foil and incubated in the dark at room temperature for two weeks, after which 50 μl of a 50:50 p-iodonitrotetrazolium violet (6 g/l): succinate (1 M) in phosphate buffer solution (PBS) (9.6 mM) was added to each well. After an additional 6 to 24-hour incubation at room temperature in the dark, plates were examined and wells that developed a pink or red colour were counted as positive. Results were expressed as most probable number of heterotrophic MPN/g of dry soil [60].

### Mineralization (activity) assays

Soil microbial activity was determined by quantifying the ability of microbes to mineralize 1-^14^C labelled acetate (specific activity 50 mCI/mmol) into ^14^CO_2_ following a previously described protocol [61], with minor modifications. Microcosms and mini-microcosms were prepared using 20 g of bulk soil and 2 g of rhizosphere soil spiked with 100,000 dpm and 50,000 dpm, respectively. For each treatment and control, i.e., unplanted, unamended mine residues, composite soil samples from five (5) randomly selected alders were tested in technical triplicates. One set of triplicates was autoclaved twice, with a 24-hour room temperature incubation between autoclavings, and used as sterile controls for the mineralization assay. Microcosms were incubated at room temperature and sampled weekly until the cumulative mineralization reached a plateau (typically within 28 days).

### DNA extraction, amplification and pyrosequencing

Total genomic DNA was extracted from all soil samples using the PowerMax® Soil DNA Isolation Kit (MoBio Laboratories, Carlsbad, CA, USA) following the manufacturer’s instructions with the addition of a final ethanol precipitation step performed by incubating samples at -20°C overnight followed by centrifugation and collection of the pellet and a brief drying step performed at room temperature in a Speedvac. The dried pellet was then resuspended in 400 μl sterile water and quantified via the PicoGreen® dsDNA quantification assay (Invitrogen, Burlington, ON).

Soil microbial community composition was investigated via sequencing of the 16S ribosomal RNA (rRNA) gene and Internal Transcribed Spacer (ITS) region of ribosomal DNA for bacteria and fungi, respectively. Partial 16S rRNA gene and ITS amplicons were produced from DNA extracts using barcoded primers and multiplexed high throughput sequencing was performed using the Ion Torrent sequencing platform [Life technologies, Grand Island, NY].

For the production of 16S rRNA gene amplicons, the universal bacterial primers F343 (5’-TACGGRAGGCAGCAG-3’) and R533 (5’-ATTACCGCGGCTGCTGGC-3’) [62] were used and PCR cycling conditions involved an initial 5 min denaturing step at 95°C, followed by 25 cycles of 30 s at 95°C, 30 s at 55°C, and 45 s at 72°C, and a final elongation step of 10 min at 72°C.

For the production of ITS amplicons, the universal fungal primers ITS1 and 58A2R [63] were used and PCR cycling conditions involved an initial 5 min denaturation step at 95°C, followed by 30 cycles of 30 s at 95°C, 30 s at 45°C, and 45 s at 72°C, and a final 10 min elongation step at 72°C.

PCRs were carried out in 0.2 ml PCR tubes each containing 10 μl of HotStartTaq Plus Master Mix (containing HotStartTaq *Plus* DNA Polymerase, PCR Buffer with 3 mM MgCl_2_, 400 μM of each nucleoside triphosphate (QIAGEN, Valencia, CA), 0.5 μl 20 mg/ml BSA, 0.5 μl of each 20 μM reverse and forward primer and 7.5 μl RNA-free water for a final volume of 20 μl. Gel purification of amplicons was performed using the illustra GFX PCR DNA and Gel Band Purification Kit (GE Healthcare, Piscataway, NJ, USA), and DNA in the purified eluate was quantified using the Quant-iT PicoGreen dsDNA assay kit (Invitrogen, Burlington, ON), pooled in an equimolar ratio and diluted to a concentration of 5 x 109 molecules/μl (approximately 1 ng/μl) for sequencing. Sequencing was performed on an Ion Torrent Personal Genome Machine™ using the Ion Xpress™ Template Kit and the Ion 314™ chip following manufacturer’s protocols. Sequence data generated from this study were deposited in the NCBI Sequence Read Archive (SRA) under accession number: SRP064667.

### Sequence quality processing, classification and downstream (OTU) analysis

Using an in-house Perl pipeline, sequences were binned by multiplex identifiers (MIDs), after which MIDs and Ion Torrent adaptor sequences were trimmed from each sequence. Sequences were then filtered using an average Q20 cutoff. If the average of 5 consecutive bases along a sequence fell below Q20, the sequence was trimmed at that point and reads of less than 75 bp were removed from downstream analysis. Taxonomic identities were assigned to sequences using the Silva and Unite databases, for bacteria and fungi, respectively, with a bootstrap of 50%. OTU analysis was performed in Mothur [64], and was used to determine Shannon diversity values and UniFrac distance between samples using a 3% dissimilarity cut-off after which classified sequence data were transformed using the Bray-Curtis distance prior to creation of a PCoA matrix [65]. A PCoA matrix of the transformed data, and ordination plots for both the taxonomic and UniFrac data were produced using the ‘vegan’ package in R (v.2.15.0, The R Foundation for Statistical Computing, Vienna, Austria).

To standardize between samples, the number of sequences representing each sample was reduced to the lowest number among all sample replicates and analysis was performed following the Mothur suggested workflow algorithms and SOPs. Samples were assembled into a single group file, and unique sequences identified using the ‘unique.seqs’ algorithm. Unique sequences were then aligned against the Silva (release 115) dataset with ‘align.seqs’. Sequences were filtered with ‘filter.seqs’, putatively noisy sequences were removed with the ‘pre.cluster’ algorithm, and possible chimeras were removed using the ‘chimera.uchime’ and ‘remove.seqs’ algorithms. A Phylip-formatted distance matrix was created using ‘dist.seqs’, and average-neighbour clustering was performed using ‘cluster.split’ and setting method = average. A shared file was then created using ‘make.shared’, and Shannon diversity was obtained using ‘summary.single’. A Phylip-formatted distance matrix was used to calculate UniFrac distances, replicates for each sample were merged with ‘merge.groups’, and weighted UniFrac values between samples were calculated using ‘unifrac.weighted’ with default parameters enabling the creation of a principle coordinate analysis (PCoA) matrix via the ‘pcoa’ command. The PCoA matrix of the transformed data and ordination plots for both the taxonomic and UniFrac data were produced using the ‘vegan’ package in R version 3.0.2 (R Foundation for Statistical Computing, Vienna, Austria).

### Statistical analysis

All statistical analyses were conducted in Mothur, JMP 11.0 (SAS Institute, Cary, NC, USA) and R version 3.0.2 (R Foundation for Statistical Computing, Vienna, Austria available at http://www.R-project.org). Differences between specific means were assessed using ANOVA and Tukey’s HSD (post-hoc) test.

## Results

### Greenhouse plant survival and growth

After 6 months of growth in the metal-contaminated mine residues *A*. *crispa* and *A*. *glutinosa* had similar survival rates with significantly lower survival in mine residues amended with woodchips (WC) relative to unamended mine residues (NT) (p<0.05) (S1 Table). The effect of plant species and the interaction of plant species and treatment on alder survival were not significant (p>0.05).

**Fig 1:**
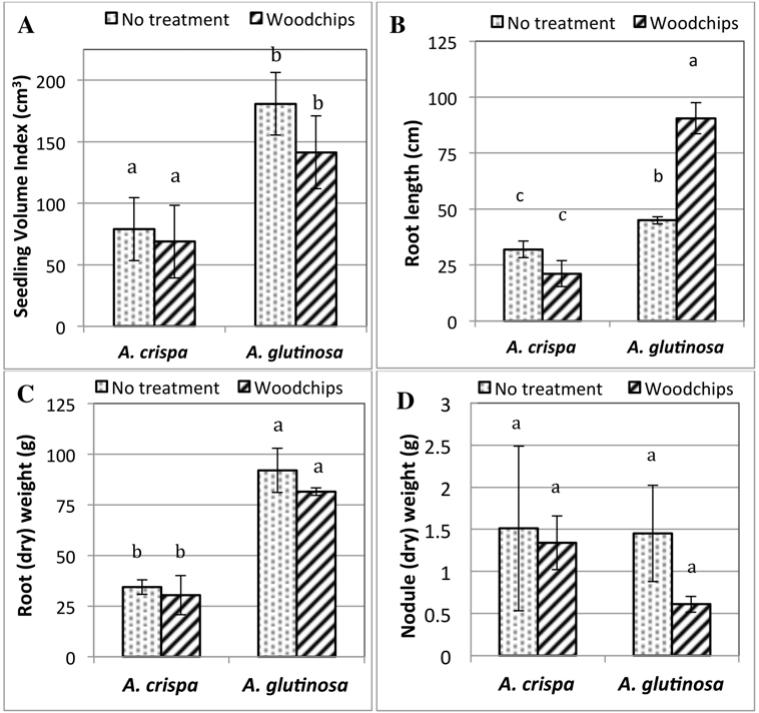
Alder growth parameters after six months of growth in mine residues. **(A) Biomass expressed as the seedling volume index (SVI); (B) Root length; (C) Root (dry) weight and (D) nodule (dry) weight.** Different letters denote significant differences of the mean as determined by Tukey’s HSD test.

Although the survival of both alders was statistically similar, *A*. *glutinosa* outperformed *A*. *crispa* in terms of growth, i.e., biomass production, as estimated by the seedling volume index (SVI) (Fig 1(A)). The addition of woodchips as an amendment did not significantly affect growth in mine residues and there were significant interacting effects of plant species and treatment on alder growth, as after 6 months of growth in mine residues, *A*. *glutinosa* had higher SVI than *A*. *crispa*, regardless of treatment (p<0.05). *A*. *crispa* also showed significantly more signs of stress than *A*. *glutinosa*, including leaf chlorosis, i.e., yellowing of leaves and browning of leaf edges. *A*. *glutinosa* had significantly larger roots than *A*. *crispa*, regardless of treatment (Fig 1(B) and 1(C)) and Tukey’s HSD test revealed that root size (length and weight) was dependent on plant species as well as the interacting effects of plant species and treatment. *A*. *glutinosa*, WC alders had significantly longer roots than NT alders (p<0.05). Root nodulation, however, specifically, root nodule (dry) weight, was statistically similar amongst all alders (p>0.05) (Fig 1(D)).

### Above-ground plant tissue metal concentrations

After six (6) months of growth in mine residues, alder above ground (leaf and stem) metal concentrations were not significantly higher than controls (healthy greenhouse alders prior to transplantation) except for manganese (p<0.05) and sodium (p<0.05) (S2 Table). Tukey’s HSD test revealed that plant tissue metal concentrations were dependent on plant species and the interaction between plant species and treatment, but not the treatment itself. In addition, *A*. *crispa* leaves contained twice as much manganese and sodium as *A*. *glutinosa* leaves, while *A*. *glutinosa* stems contained twice as much manganese as *A*. *crispa* leaves (p<0.05).

### Soil quality

**Fig 2:**
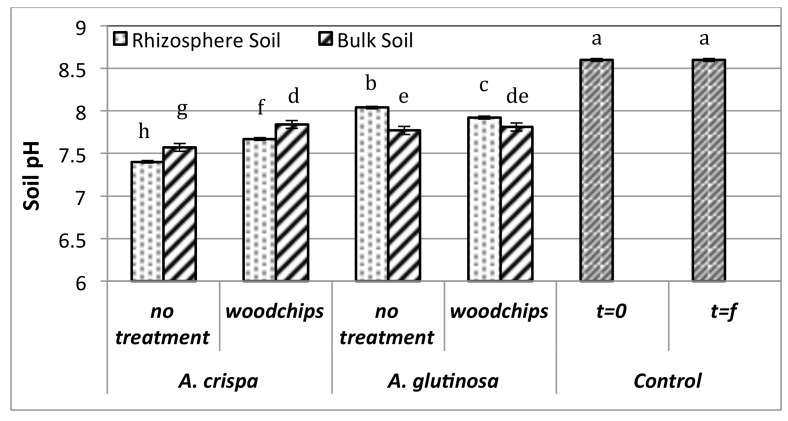
Alder rhizosphere and bulk soil pH after six months of growth in mine residues. Letters above columns denote significant differences between means (p<0.05), as determined by Tukey’s HSD test.

Plant colonization positively influenced soil pH as both the rhizosphere (RZ) and bulk (BK) soil of planted pots had significantly lower pH relative to unplanted controls (pH 8.6) (p<0.05) **(**Fig 2). Tukey’s HSD test revealed that soil pH was dependent on plant species, soil fraction sampled (i.e., whether rhizosphere or bulk soil), whether or not an amendment (woodchips) was added and the interaction of these factors. Improvements in soil structure and water retention were also observed in planted pots as alders established in mine residues, enhancing soil stability and improving soil fertility.

Total extractable metals in alder soils were highly dependent on the soil fraction analyzed and not plant species, nor treatment. RZ and BK soils had significantly lower nickel, chrome, barium and cobalt concentrations relative to unplanted controls (p<0.05). Except for sodium and copper, RZ and BK soils did not have significantly higher metal concentrations than unplanted controls. Further, RZ had significantly lower sodium and manganese and higher aluminum and copper than BK soil (p<0.05) (Table 1).

**Table 1:** Alder rhizosphere and bulk soil metal concentrations after six (6) months of growth.

			Rhizosphere Soil				Bulk Soil				
			*Alnus crispa*		*Alnus glutinosa*		*Alnus crispa*		*Alnus glutinosa*		
Metal (ppm)	Maximum Acceptable Limits	Unplanted Control	NT	WC	NT	WC	NT	WC	NT	WC	LDR
**Aluminum (Al)**	n/a	17 000 ± 80	19 000 ± 168	20 000 ± 154	19 000 ± 127	19 000 ± 106	18 000 ± 121	18 000 ± 93	16 000 ± 89	18 000 ± 104	20
**Manganese (Mn)**	2200	690 ± 9	680 ± 10	650 ± 14	770 ± 12	710 ± 15	860 ± 11	800 ± 8	770 ± 8	760 ± 15	2
**Sodium (Na)**	n/a	77 ± 6	**230 ± 7	**320 ± 12	**190 ± 12	**180 ± 9	**300 ± 17	**260 ± 9	**300 ± 8	**450 ± 15	2
**Copper (Cu)**	500	55 ± 3	**80 ± 4	**84 ± 4	**110 ± 9	**130 ± 9	** 84 ± 2	**90 ± 6	67 ± 3	67 ± 3	5
**Zinc (Zn)**	1500	51 ± 3	68 ± 2	71 ± 5	70 ± 5	73 ± 4	71 ± 3	63 ± 6	72 ± 3	72 ± 3	40
**Barium (Ba)**	2000	79 ± 3	*35 ± 1	58 ± 17	*21 ± 3	*33 ± 5	*14 ± 2	14 ± 2	*12 ± 2	*15 ± 1	2
**Chromium (Cr)**	800	110 ± 3	*25 ± 2	*30 ± 5	*20 ± 4	*26 ± 2	*19 ± 2	*18 ± 1*	*17 ± 4	*19 ± 1	10
**Nickel (Ni)**	500	45 ± 4	*17 ±1	*23 ± 4	*13 ± 4	*17 ± 2	*12 ± 2	*12 ± 0	*11 ± 1	*12 ± 0	1
**Cobalt (Co)**	300	16 ± 1	*15 ± 3	*16 ± 3	*12 ± 1	*12 ± 0	*11 ± 0	*11 ± 1	*11 ± 0	*11 ± 0	2

Asterisks (* and **) denote significant decreases and increases, respectively, in extractable soil metal concentration differences (p<0.05). (NT = no treatment, WC = woodchips). Generic (evaluation) criteria based on the maximum acceptable limits for commercial sites not located in a residential area, and for industrial sites as described in Appendix 2 of the “Soil protection and contaminated sites rehabilitation policy” (Ministère de l’Environnement et de la Faune, 1998 [66]).

### Soil microbial community analysis

#### Microbial enumeration

**Fig 3:**
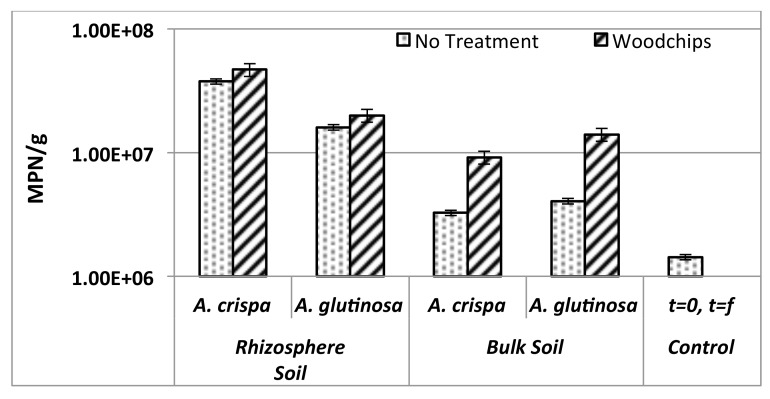
THB population enumeration for alder rhizosphere, alder bulk and control soils initially, i.e. at t = 0 (healthy greenhouse alders) and at t = f (6 months of growth in mine residues) with and without the addition of woodchips as a soil amendment.

Total heterotrophic bacterial (THB) populations were enumerated using the MPN method and results are illustrated in Fig 3. Soil THB populations were shown to be dependent on plant species and the type of soil fraction examined and not on treatment, nor the interaction of these factors.

RZ and BK soils had significantly larger THB populations (one to almost two orders of magnitude higher) than unplanted controls. Ac RZ soil had significantly higher numbers of THB than Ag RZ, while Ag BK soil had more THB relative to Ac BK. It should be noted that the initial RZ and BK measurements correspond to those of the alders just prior to transplantation at t = 0, i.e., healthy alders grown in peat under optimal greenhouse conditions immediately prior to transplantation into mine residues.

#### Microbial activity (mineralization) assays

**Fig 4:**
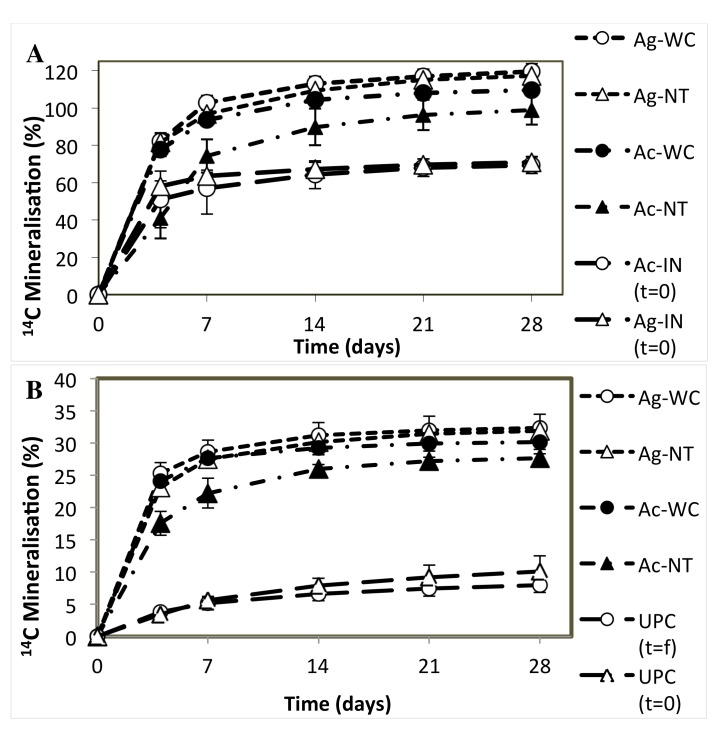
**Alder rhizosphere (A) and bulk (B) soil** ^**14**^**C mineralization activity after six months of growth in mine residues relative to initial and unplanted controls.** (Ag = *A*. *glutinosa*, Ac = *A*. *crispa*, WC = woodchips, NT = no treatment, IN = initial (healthy greenhouse alder seedlings prior to transplantation).

Changes in soil microbial mineralization activity were found to be dependent on the soil fraction sampled. For rhizosphere soil only, plant species and treatment were also found to significantly affect mineralization. Alder rhizosphere soils exhibited up to four times more mineralization activity than bulk soils (p<0.05); and both alder bulk and rhizosphere soils had significantly higher activity relative to initial mineralization rates (p<0.05) (Fig 4). Mineralization rates in final alder rhizospheres were, on average, 71% higher than initial (t = 0) healthy greenhouse alder rhizospheres. While there were no statistically significant differences in mineralization potential of Ac and Ag rhizospheres initially, i.e., prior to transplantation into mine residues, final Ag rhizospheres had on average 15% higher mineralization than Ac rhizosphere (Fig 4A), regardless of the treatment applied. Ac-WC rhizosphere was found to have (10%) higher microbial activity than Ac-NT: this effect was not seen in Ag rhizosphere.

#### Microbial community composition, diversity and relative abundance

**Fig 5:**
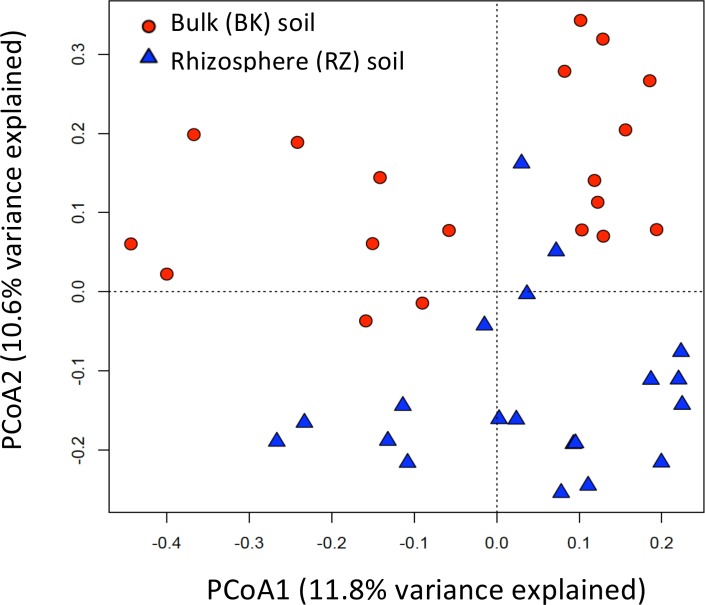
Principal coordinate analysis (PCoA) plot illustrating ecological relatedness of RZ and BK soil microbial communities with 22.4% variance explained.

After sequence filtering, a total of 158,642 usable reads were obtained, with an average of 2,644 reads per sample replicate. Shannon Diversity indices, calculated using a 3% dissimilarity cut-off, were higher in planted soils relative to unplanted controls, with rhizosphere soils significantly more diverse than bulk soils (p<0.05) (S3 Table). Weighted principal coordinate analysis (PCoA) ordination revealed that microbial community composition was highly dependent on soil type, i.e., the soil fraction analyzed (p<0.05) as samples grouped together almost exclusively based on whether the soil originated from alder rhizosphere (RZ) or bulk (BK) soil (Fig 5).

**Fig 6:**
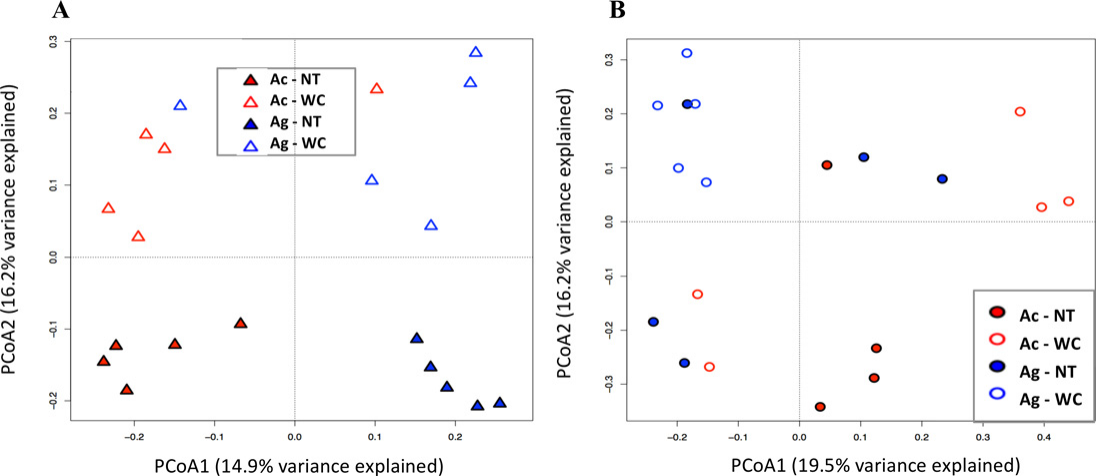
**PCoA plot illustrating the community similarity, i.e., relatedness, of (A) rhizosphere and (B) bulk soil microbial communities with 31.1% and 35.7% variance explained, respectively.** (Ac = *A*. *crispa*, Ag = *A*. *glutinosa*, NT = no treatment, WC = woodchips).

For alder RZ, average weighted UniFrac distances between different samples was higher than that amongst sample replicates (p<0.05), i.e., samples belonging to the same treatment formed clearly defined clusters, except for one outlier each for Ac-WC and Ag-WC RZ. Except for Ag-WC, well-defined clusters were not observed in BK soils as average weighted UniFrac distances between soils within the same treatment were higher in BK versus RZ soil (p<0.05) and PCoA ordination plots showed that microbial community composition spread further across both axes in BK soils than RZ soils (Fig 6).

**Fig 7:**
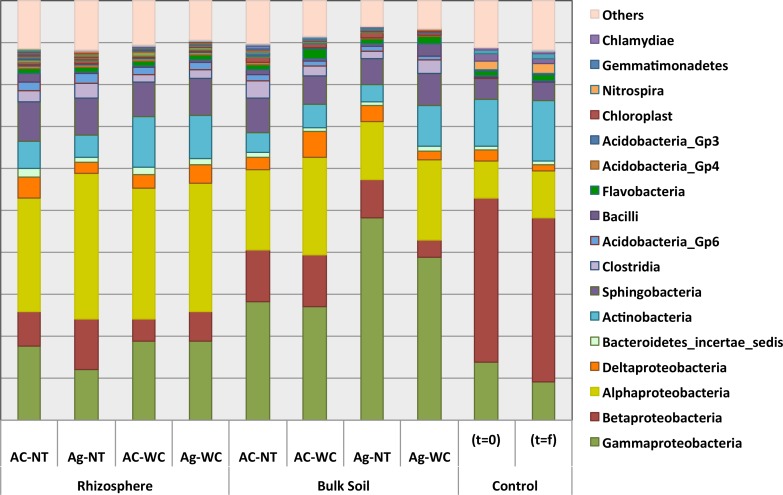
Alder rhizosphere and bulk soil class-level bacterial community profiles after six (6) months of growth, relative to unplanted controls. (Ac = *A*. *crispa*, Ag = *A*. *glutinosa*, NT = no treatment, WC = woodchips). Asterisks (*) denote orders designated incertae sedis.

The majority (roughly 79%) of bacterial sequences retrieved belonged to the *Alphaproteobacteria*, *Betaproteobacteria*, *Deltaproteobacteria*, *Gammaproteobacteria*, *Actinobacteria* and *Sphingobacteria* bacterial classes. Fig 7 illustrates rhizosphere and bulk soil bacterial community composition after six (6) months of growth in mine residues. Soil bacterial community compositions of planted plots were found to be significantly different from unplanted pots (p<0.05) although no significant differences amongst the different treatments and/or species were observed, except for the bulk soil of *A*. *glutinosa* pots amended with woodchips (BK Ag-WC).

The relative abundances of *Alphaproteobacteria* and *Betaproteobacteria* in soils was significantly correlated with whether or not pots were planted as there were significant decreases and increases in *Betaproteobacteria* and *Alphaproteobacteria*, respectively (p<0.05) in both alder RZ and BK soil. In mine residues at the start of the experiment (t = 0) and unplanted soil after the six-month incubation period (t = f), *Betaproteobacteria* and *Alphaproteobacteria* comprised approximately 39% and 10% of classified sequences, respectively. After six (6) months of growth in mine residues *Betaproteobacteria* accounted for, on average, 10% of sequences retrieved from all BK and RZ samples, compared to 25% for *Alphaproteobacteria*.

**Fig 8:**
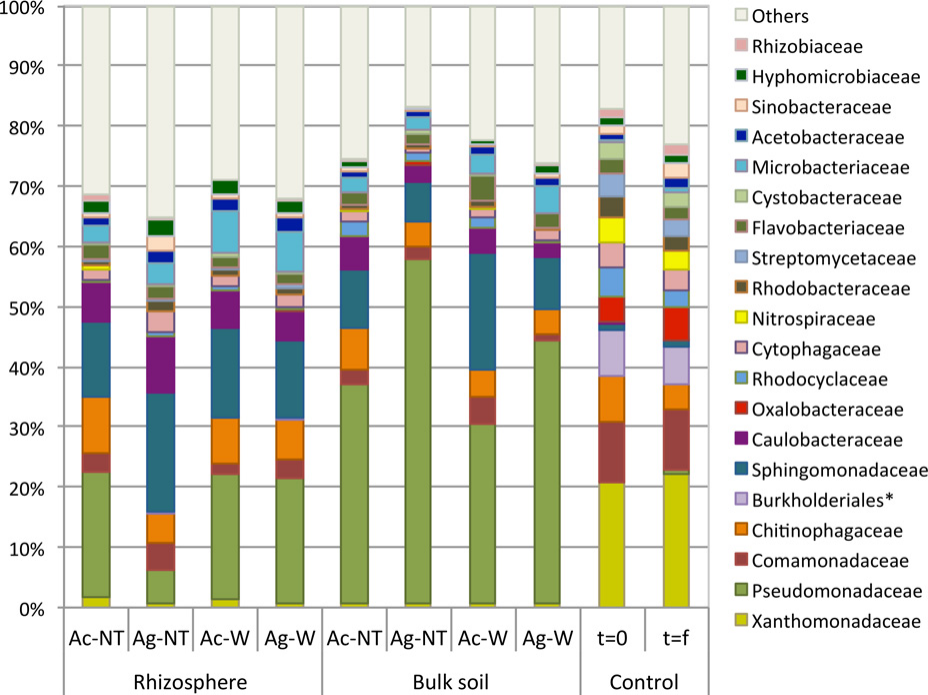
Alder rhizosphere and bulk soil family-level bacterial community profiles after six (6) months of growth, relative to unplanted controls. (Ac = *A*. *crispa*, Ag = *A*. *glutinosa*, NT = no treatment, WC = woodchips). Asterisks (*) denote orders designated incertae sedis.

In the t = 0 and t = f control soils, the bacterial families *Xanthomonadaceae*, *Comamonadaceae* and *Burkholderials_incertae_sedis* comprised approximately 31% of sequences (Fig 8). Significant decreases in their abundances were observed after six (6) months of alder growth, as these taxa only comprised approximately 3% of sequences (p<0.05). *Sphingomonadaceae* (*Sphingobium* and *Sphingomonas* genera), *Pseudomadaceae (Pseudomonas* and *Azotobacter* genera) and *Caulobacteraceae* (*Brevundimonas* and *Caulobacter* genera) were instead the dominant bacterial families in both BK and RZ soils, comprising 8–12%, 2–40% and 1–5% of sequences, respectively.

**Fig 9:**
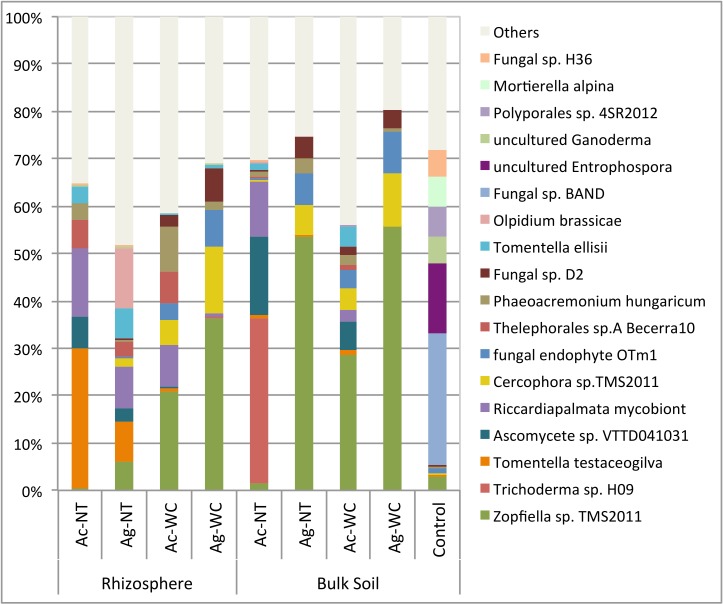
Alder rhizosphere (RZ) and bulk (BK) soil fungal (ITS) community profiles after six (6) months of growth in mine residues relative to initial (t = 0) controls. (Ac = *A*. *crispa*, Ag = *A*. *glutinosa*, NT = no treatment, WC = woodchips).

After sequence filtering, a total of 264,564 usable reads were obtained, with an average of 4,409 reads per sample replicate. Fig 9 illustrates the changes in fungal community structure during the course of the experiment. In unplanted mine residues at t = f, i.e., after a six (6) month incubation period, six (6) main taxonomic groups (uncultured *Entrophospora*, uncultured *Ganoderma*, *Mortierella alpinda*, Fungal sp. *BAND*, Fungal sp. *H36* and *Polyporales* sp. *4 SR-2012*) dominated fungal communities, comprising more than 41% of classified fungal sequences. After six (6) months of alder growth, shifts in relative abundance were observed and these taxa comprised less than 1% of classified sequences from all RZ and BK soils combined (Fig 7). Alder BK and RZ soil fungal community composition was found to be significantly dependent on plant species, amendment and the interaction between these factors (p<0.05) while all treatments were found to be significantly different from each other as well as unplanted controls (p<0.05). Significant increases in the abundance of several important fungal species in RZ and BK soil communities were observed following alder growth in mine residues with the following fungal species comprising 40% to 85% of sequences; *Zopfiella* sp. *TMS-2011* (28%), *Trichoderma* sp. *H09* (14%), *Tomentella testaceogilva* (13%), *Ascomycete* sp. *VTTD041031* (7%), *Arthrobotrys scaphoides* (6%), uncultured *Sebacina mycobiont* of *Riccardiapalmata* (6%), *Cercophora* sp. *TMS-2011* (5%), Fungal endophyte *OTm-1* (4%), *Ramophialophora humicola* (4%), *Thelephorales* sp. *A*. *Becerra 10* (3%), *Phaeoacremonium hungaricum* (3%), *Fusarium* sp. *JJP-2009a* (2%).

## Discussion

### Performance of *Frankia*-alder symbionts in Val d’Or gold mine residues

Mine residues are challenging substrates for plant growth and studies have emphasized the importance of *Frankia* inoculation for adequate plant root nodule formation, a crucial feature for tolerating such harsh, nutrient-poor environments [20; 67–69]. Previous revegetation trials on an oil sands tailings site showed that actinorhizal alders grew better than uninoculated plants in terms of plant biomass production and root development [16] and that it is critical to inoculate alders prior to transplantation into tailings so that nodules benefit the plant instead of requiring energy for their development. Studies have also demonstrated the difficulty of mycorrhizal growth in mine tailings as such nutrient-poor conditions are not optimal for mycorrhizal development [9; 70–72]. Dual inoculation with *Frankia* and ECM fungi has had variable impact (slightly negative, neutral, or highly positive) on alder (*A*. *glutinosa* and *A*. *crispa*) growth and performance, and on soil microorganisms when compared to *Frankia* inoculation alone [16, 20, 25, 70, 73].

In this greenhouse experiment, both actinorhizal alder species were able to establish and grow in gold mine waste rock mixed with fine tailings, from Val d’Or, Quebec. After six (6) months of growth both *A*. *glutinosa* and *A*. *crispa* had significantly lower survival, but not growth rates, in mine residues amended with woodchips relative to unamended pots. *A*. *glutinosa* outperformed *A*. *crispa* in terms of biomass production as estimated by the seedling volume index (SVI). A finding that may be attributed to the fact that *A*. *glutinosa* is a shade tolerant tree species, while *A*. *crispa* is a shrub species requiring abundant sunlight. *A*. *crispa* also showed significantly more signs of stress than *A*. *glutinosa*, which included yellowing of leaves and browning of leaf edges, illustrating species-specific plant growth responses to mine substrates as seen in other studies [74]. While alder root nodulation, specifically the number and weight of root nodules, was statistically similar in both alders, *A*. *glutinosa* had significantly larger and longer roots compared to *A*. *crispa*, leading to a greater impact on rhizosphere soil and microorganisms. This is an important result since the efficiency of phytoremediation is largely dependent on root zone size and root-soil microbe interactions.

When mine residues were planted with alders, soil metal concentrations, except for sodium and copper, were reduced. Improvements in soil structure and fertility were observed, including improved water retention, likely due to increased soil stability from alder root structures. Variable increases in bioavailable soil sodium amongst treatments, particularly in bulk soils, suggests that alders may have needed more time to establish in mine residues in order to effect changes in soil sodium and consequently soil salinity.

Zinc, copper, cobalt and molybendenum are some essential plant micronutrients important in non-leguminous plants bearing nitrogen-fixing root nodules. Total extractable copper concentrations increased in both rhizosphere and bulk soils following 6 months of alder growth in metal-contaminated mine residues, especially for *A*. *glutinosa*, similar to previous reports of alders growing in heavily polluted soils [75–77]. Efficient nitrogen fixation is dependent on the presence of adequate copper in root nodules. Alders grown in reduced copper conditions exhibited low aboveground biomass and low nitrogen content [75]. There is also strong evidence that alders, *A*. *incana* and *A*. *glutinosa* in particular, are copper excluders, i.e. plants which may actively take up and accumulate copper in roots and nodules but avoid transport of these metal ions to shoots and leaves [76, 77]. This is partially corroborated by the finding that, in this, experiment final copper concentrations in *A*. *glutinosa* aboveground tissue (leaves and stems) were not found to be significantly different from controls, and only slightly higher than controls in *A*. *crispa* stems.

Alders decreased soil pH significantly to near neutral conditions (to pH 7.5), even in bulk soils, improving soil nutrient bioavailability [78]. *A*. *crispa* effected greater reductions in soil pH than *A*. *glutinosa*, despite the fact that *A*. *glutinosa* had greater root biomass, particularly in unamended and rhizosphere soils, likely due to effects of rhizodeposition (root turnover and exudates). This is particularly important as it demonstrated that the use of amendments including woodchips and fertilizer are not necessary to significantly improve soil quality during the revegetation of metal contaminated mine residues, contrary to previous reports [33–34; 70–81]. For *A*. *glutinosa*, bulk soils had a significantly lower pH than rhizospheres demonstrating the significant impact actinorhizal alder growth can have on total bulk mine residues, not just rhizosphere and adjacent soils. Improvements in soil texture and structure, pH and soil metal concentrations effectuated by actinorhizal alders may enable the establishment of more sensitive plant species, increasing plant diversity and potentially restoring ecosystem function in mine-degraded environments [82].

After six months of growth in mine residues, metal concentrations in aboveground biomass (leaves and stems) of planted alders were not significantly higher than those of healthy alders grown in optimal (greenhouse) conditions prior to transplantation into mine residues, except for manganese, sodium and aluminum in *A*. *crispa*. The addition of woodchips as a soil amendment to mine residues planted with *A*. *glutinosa* reduced sodium content of leaves and stems to below detectable limits. Similar results were not observed for *A*. *crispa*, both similar and in contrast to previous reports on alder revegetation of oil sands tailings [16, 83], demonstrating site- and species-specific responses to alder growth in metal contaminated mine soils. All alder aboveground metal concentrations were found to be well below plant tissue and domestic animal toxicity limits based on mean values of toxic levels of accumulated metals in agricultural crops [84], and maximum tolerable levels for grazing animals [85], respectively. Thus, the use of actinorhizal alders (*A*. *crispa* and *A*. *glutinosa*) in the revegetation of mine residues did not constitute a toxicity threat, i.e pose health risks to fauna potentially grazing on them.

### Microbial community dynamics

Plant growth and soil physiochemical parameters have been extensively used in the evaluation of success of phytoremediation strategies [35]. However, the sensitivity, rapid response, and integrative character of biological indicators of soil health, namely those related to the size, activity, and diversity of soil microbial communities, make them invaluable tools for assessing the efficiency and impacts of metal phytoremediation [33–34]. The planting of alders in mine tailings was found to positively stimulate microbial populations as corroborated by increases in bacterial density and carbon (acetate) mineralization potential, i.e., metabolic activity. Microbial enumeration of total heterotrophic bacteria demonstrated significant increases in the population densities of alder rhizosphere soils relative to both bulk soils and unplanted controls. Bulk soil microbial counts were up to one order of magnitude higher than unplanted controls, and the use of woodchips as an amendment significantly increased bulk soil THB populations to levels comparable to or higher than initial, healthy (greenhouse-produced) alders prior to transplantation into mine residues, demonstrating alder-induced stimulation of the entire soil microbial population, not only the rhizosphere fraction. This is contrary to reports from previous field and greenhouse trials in both oil sands process affected material (OSPM) and metal-mine soils of similar pH (pHs 7–8) [9; 86, 87], illustrating the site-specific nature of alders’ below-ground zone of influence and effects on soil microbial communities within, and outside of, the root zone.

Acetate mineralization assays revealed that mine bulk soil microbial communities were not only larger, but also significantly more metabolically active (six times more active) than unplanted controls following the planting and establishment of alders in mine residues. Alder rhizosphere soils had up to four times greater activity than bulk soils and up to two times greater activity than levels recorded in healthy greenhouse alders prior to transplantation, with complete (100%) mineralization in some samples, including unamended alder pots. This suggests that the microbial community may have benefitted from external sources of organic carbon added to soils, including root exudates and the greenhouse (peat) substrate transplanted into the mine residues with plants. *A*. *crispa* had a greater impact on increasing rhizosphere microbial biomass than *A*. *glutinosa*, with microbial population densities in these samples surpassing those in healthy (greenhouse) alders grown in peat soil, demonstrating that the two different species can have similar and differing effects on soil microbial population dynamics, corroborating findings presented in other reports [88–89]. The use of woodchips did not significantly influence soil microbial carbon mineralization patterns and alder-colonized mine residues were able to promote and sustain soil microbial activity without the addition of a fertilizer-type substrate. This is similar to findings of *Frankia*-alder growth on oil sands tailings [16], although our use of fertilizer did increase bacterial density in bulk soils.

Pyrosequencing has been used to characterize complex microbial communities [90] and in biomonitoring during bioremediation or phytoremediation trials [91–97]. In this greenhouse study, pyrosequencing revealed that the microbial community composition in all alder rhizosphere and bulk soils were distinct from each other and unplanted controls, except for the rhizospheres of woodchip-amended pots which had highly similar microbial communities despite the fact that two different alder species were growing on them. The rhizosphere is known to be a nutrient rich environment, which stimulates the activity and diversity of microbial communities [98] and the diverse and specific root exudates produced by different plant species and their utilization by microorganisms can exert dissimilar selective pressures on microbial communities [87, 99]. Although fertilizer use, i.e., the addition of woodchips, did not significantly influence alder survival and growth, it did significantly impact indigenous microbial communities. The findings of this study suggest that soil amendments can have an equivalent, or greater influence than plant species on soil microbial community composition and structure, particularly in challenging, nutrient-poor environments such as mine-degraded land. The use of soil amendments in site rehabilitation must therefore consider the needs of actinorhizal alders capable of initiating the process, the environmental cost of amendment application including substantial fossil fuel consumption use during manufacturing and, most importantly, the overall goal; typically the re-establishment of a biologically diverse, functional and self-sufficient ecosystem.

Principal coordinate analysis (PCoA) also revealed that microbial communities from rhizosphere soil samples clustered together and separately from bulk soil samples, suggesting differences in their microbial population composition as observed [16, 100]. Rhizosphere soil samples from the same treatment (plant species and amendment type) were more ecologically similar than those from different treatments, i.e., the dissimilarity of microbial communities within groups of treatments was smaller than the dissimilarity among different treatment groups. This effect was similar, although less pronounced, in bulk soils.

As pH is a fundamental predictor of soil bacterial community structure [52], even at the continental level [36], shifts in soil bacterial community composition following alder growth and in mine soils were predicted as alders changed mine waste rock from basic to close to neutral (pH 7.5 or lower). Differences in microbial community composition amongst samples were observed, despite the fact that almost all treatments had statistically similar bacterial population densities and carbon mineralization rates, illustrating the major and complex role of plant species and treatment combined, in determining microbial community composition primarily through rhizodeposition (root turnover and exudates) [101]. Microbial community profiles showed a high diversity in rhizosphere soils relative to unplanted controls with similar Shannon Diversity for all rhizosphere and bulk soil treatments. The use of woodchips as a mine residue amendment increased the Shannon diversity of rhizosphere, but not bulk soil microbial communities.

The majority (up to 79%) of classified bacterial sequences belonged to *Alphaproteobacteria*, *Betaproteobacteria*, *Deltaproteobacteria*, *Gammaproteobacteria*, *Actinobacteria* and *Sphingobacteria*, roughly corresponding to results of previous studies [36, 52, 102–104]. In addition, *Frankia*-alder soil bacterial communities shifted from *Betaproteobacteria*- to *Alphaproteobacteria*-dominated, corroborating previous reports of a 3:1 *Alphaproteobacteria* to *Betaproteobacteria* ratio in healthy soil and 2:1 ratio in neutral mine drainage [52] and suggesting that the abundance of *Alphaproteobacteria*, or at least, the ratio of the relative abundances of *Alphaproteobacteria* to *Betaproteobacteria* may be a useful indicator species for assessing soil health status. Previous studies have reported enrichments of *Alphaproteobacteria*, *Gammaproteobacteria* and *Actinobacteria* in the rhizosphere where root exudates increase carbon availability [52, 100, 105, 106] Despite the fact that *Betaproteobacteria* and *Bacteriodetes* have also been previously shown to be positively correlated with carbon mineralization across a range of soils [105], Val d’Or mine soils (i.e., waste rock and tailings) did not suffer from a loss of mineralization potential following alder plantation and a shift away from *Betaproteobacteria*-dominated bacterial communities. In fact, the opposite was true suggesting that a shift in dominant taxa, possibly towards *Alphaproteobacteria-*dominated communities likely due to selective effects of plant root exudates on soil microbial community composition as described by previous authors [107], may be responsible for the increase in mineralization capacity following alder plantation.

*Alphaproteobacteria* and *Actinobacteria* dominated all planted samples (comprising up to 70% of sequences) and significant increases in the abundance of several important bacterial genera including the contaminant degraders *Sphingomonas*, *Sphingobium* and *Pseudomonas*, nitrogen fixing bacteria of the *Azotobacter*, *Mesorhizobium*, *Rhizobium* and *Pseudomonas* genera and the metal sequestering bacteria *Brevundimonas* and *Caulobacter*, were observed. These shifts in microbial community structure in alder rhizospheres relative to unplanted controls suggest that initial soil conditions on site, that favoured the growth of low-nutrient-adapted microorganisms (oligotrophs), changed during the establishment of alders, demonstrating the positive effect of actinorhizal alders on microbial community structure, function and ecosystem health.

Globally, approximately 60 species of ECM fungi have been identified as mycorrhizal symbionts of alders and associations have been shown to be dependent on multiple factors including host evolutionary history, mean annual temperature and precipitation, pH and soil calcium concentration [108]. In this study, rhizosphere (RZ) and bulk (BK) soil fungal community compositions of all treatments significantly differed from each other as well as unplanted controls following six months of alder growth in mine residues. Although increases in the relative abundance of common alder-associated ECM fungal taxa such as *Tomentella and Thelephorales* [108–109] and ECM fungal taxa such as *Fusarium*, previously isolated from heavy metal polluted waterways [110] were observed in most treatments, no significant trends emerged. Differential plant growth responses to various ECM fungi have been observed in numerous inoculation and greenhouse studies, however, few studies have investigated naturally occurring ECM symbiosis in plants grown under field conditions [109] demonstrating the need for such studies.

Further examination of microbial community dynamics over longer durations of phytostabilization and at later successional stages on mine residues and other degraded lands may provide a clearer understanding of tripartite (plant-bacteria-fungi) interactions in these environments. This knowledge will enable the development of more efficient and effective land reclamation strategies.

## Conclusions

In this study, pyrosequencing was successfully used to directly evaluate tripartite (plant-bacteria-fungi) interactions during the phytostablization of mine tailings. Treatment-specific shifts in microbial community composition following alder establishment highlight the complex nature of plant-microbe interactions and effects on the soil microbial community structure and composition.

It was shown that revegetation and phytostablization of metal contaminants from gold mine waste rock could be successfully achieved using actinorhizal alders without the use of amendments. Overall, alders were capable of establishing and performing well on mine residues, producing significant biomass. Growth of alders in mine waste rock significantly lowered metal toxicity (via reduced bioavailability) and improved soil quality while positively impacting indigenous soil microbial community as illustrated by increases in soil microbial density and activity. Significant increases in the abundance of known alder-associated ECM fungi species and bacterial taxa involved in important ecosystem processes such as nitrogen fixation, contaminant degradation and metal sequestration were observed. In addition, bacterial communities in planted mine residues experienced marked increases and decreases in *Alphaproteobacteria* and *Betaproteobacteria*, respectively, suggesting that *Alphaproteobacteria* may be a useful biological indicator of soil health.

There is still a need to improve phytostabilization techniques, particularly in Canada, where mining is important to our economy and the average lifetime of Canadian mines is under 20 years. Our findings suggest that capitalizing on native plants’ tripartite associations, i.e. plants’ natural symbiotic associations may be a superior, low-cost alternative to other in-situ bioremediation techniques. This study also highlights the need to evaluate the effect and relevance of using soil amendments when actinorhizal plants are to be used as drivers in land reclamation practices.

## Supporting Information

S1 Table Alder survival rates after six months of growth in mine residues. Asterisks (*) denote significant differences between treatments.
(DOCX)

S2 Table Plant metal concentrations for Ag (A) and Ac (B) above-ground biomass (leaves and stems). Asterisks (*) denote significant differences (p<0.05) relative to t = 0, i.e., IN alders. (WC = woodchips, NT = no treatment, IN = initial, i.e. healthy greenhouse alder seedlings prior to transplantation).
(DOCX)

S3 Table Shannon diversity and Simpson Index estimates for BK and RZ soil samples after six months of growth in mine residues. (DOCX)
