# Supplementary material for: Actinorhizal Alder Phytostabilization Alters Microbial Community Dynamics in Gold Mine Waste Rock from Northern Quebec: A Greenhouse Study
Source: PLoS One. 2016 Feb 29;11(2):e0150181. doi: 10.1371/journal.pone.0150181 (PMC4771167; doi:10.1371/journal.pone.0150181)
Supplement: S1 Table — Asterisks (*) denote significant differences between treatments. (DOCX) [file pone.0150181.s001.docx]

**S1 Table. Alder survival rates after six months of growth in mine residues.**

|  | ***A. crispa* (%)** | ***A. glutinosa* (%)** |
| --- | --- | --- |
| **No treatment** | 100.0 ± 0.00 | 100.0 ± 0.00 |
| **Woodchips** | 90.0 ± 2.89 | 90.0 ± 2.89 |

Asterisks (*) denote significant differences between treatments.
