# Supplementary material for: Actinorhizal Alder Phytostabilization Alters Microbial Community Dynamics in Gold Mine Waste Rock from Northern Quebec: A Greenhouse Study
Source: PLoS One. 2016 Feb 29;11(2):e0150181. doi: 10.1371/journal.pone.0150181 (PMC4771167; doi:10.1371/journal.pone.0150181)
Supplement: S2 Table — Plant metal concentrations for Ag (A) and Ac (B) above-ground biomass (leaves and stems). Asterisks (*) denote significant differences (p<0.05) relative to t = 0, i.e., IN alders. (WC = woodchips, NT = no treatment, IN = initial, i.e. healthy greenhouse alder seedlings prior to transplantation). (DOCX) [file pone.0150181.s002.docx]

**S2 Table. Plant metal concentrations for Ag (A) and Ac (B) above-ground biomass (leaves and stems).**

| A | ***A. glutinosa* (Ag)** | | | | | |
| --- | --- | --- | --- | --- | --- | --- |
|  | **Leaves** | | | **Stems** | | |
| **Metal (ppm)** | **IN** | **NT** | **WC** | **IN** | **NT** | **WC** |
| **Manganese (Mn)** | **43 ± 8** | ***180 ± 9** | ***170 ± 10** | **71 ± 7** | ***300 ± 11** | ***320 ± 13** |
| **Sodium (Na)** | **58 ± 9** | ***51 ± 8** | ***<40** | ***240 ± 0** | ***90 ± 7** | ***<40** |
| **Aluminium (Al)** | **<20** | **<20** | **<20** | **<20** | **<20** | **<20** |
| **Zinc (Zn)** | **22 ± 2** | **26 ± 2** | **35 ± 3** | **31 ± 5** | **28 ± 6** | **40 ± 5** |
| **Barium (Ba)** | **5 ± 0** | ***12 ± 1** | ***12 ± 0** | **6 ± 0** | **8 ± 0** | **8 ± 0** |
| **Copper (Cu)** | **6 ± 0** | **7 ± 0** | **7 ± 1** | **9 ± 1** | **10 ± 2** | **8 ± 2** |
| **Molybdenum (Mo)** | **3 ± 1** | **<1** | **<1** | **15 ± 4** | **3 ± 1** | **2 ± 0** |
| **Nickel (Ni)** | **1 ± 0** | **1 ± 0** | **<1** | **3 ± 4** | **2 ± 2** | **2 ± 2** |
|  | | | | | | |
| B | ***A. crispa* (Ac)** | | | | | |
|  | **Leaves** | | | **Stems** | | |
| **Metal (ppm)** | **IN** | **NT** | **WC** | **IN** | **NT** | **WC** |
| **Manganese (Mn)** | **116 ± 11** | ***510 ± 17** | ***470 ± 15** | **35 ± 0** | ***180 ± 8** | ***200 ± 12** |
| **Sodium (Na)** | **370 ± 75** | ***240 ± 53** | ***550 ± 85** | **520 ± 15** | ***93 ± 12** | ***300 ± 16** |
| **Aluminum (Al)** | **32 ± 10** | **44 ± 7** | **50 ± 9** | **<20** | **22 ± 5** | **<20** |
| **Zinc (Zn)** | **37 ± 2** | **27 ± 2** | **20 ± 2** | **28 ± 1** | **24 ± 0** | **24 ± 0** |
| **Barium (Ba)** | **12 ± 2** | **9 ± 1** | **11 ± 3** | **8 ± 0** | **7 ± 0** | **9 ± 1** |
| **Copper (Cu)** | **9 ± 2** | **7 ± 1** | **6 ± 1** | **5 ± 0** | ***20 ± 5** | ***11 ± 3** |
| **Molybdenum (Mo)** | **8 ± 0** | **4 ± 0** | **4 ± 0** | **15 ± 2** | ***4 ± 1** | ***3 ± 1** |
| **Nickel (Ni)** | **3 ± 1** | **1 ± 0** | **2 ± 1** | **1 ± 0** | **4 ± 1** | **2 ± 1** |

Asterisks (*) denote significant differences (p<0.05) relative to t=0, i.e., IN alders. (WC = woodchips, NT = no treatment, IN = initial, i.e. healthy greenhouse alder seedlings prior to transplantation).
