# Supplementary material for: Actinorhizal Alder Phytostabilization Alters Microbial Community Dynamics in Gold Mine Waste Rock from Northern Quebec: A Greenhouse Study
Source: PLoS One. 2016 Feb 29;11(2):e0150181. doi: 10.1371/journal.pone.0150181 (PMC4771167; doi:10.1371/journal.pone.0150181)
Supplement: S3 Table — (DOCX) [file pone.0150181.s003.docx]

**S3 Table. Shannon diversity and Simpson Index estimates for BK and RZ soil samples after six months of growth in mine residues.**

| **Soil fraction** | **Plant species** | **Amendment** | **Shannon Diversity** | **Simpson's Index** |
| --- | --- | --- | --- | --- |
| **Rhizosphere** | ***A. crispa*** | **No treatment** | 4.90 ± 0.07 | 0.98 ± 0 |
|  |  | **Woodchips** | 4.92 ± 0.05 | 0.98 ± 0 |
|  | ***A. glutinosa*** | **No treatment** | 4.90 ± 0.08 | 0.98 ± 0 |
|  |  | **Woodchips** | 4.98 ± 0.07 | 0.99 ± 0 |
| **Bulk Soil** | ***A. crispa*** | **No treatment** | 4.65 ± 0.06 | 0.98 ± 0 |
|  |  | **Woodchips** | 4.26 ± 0.29 | 0.94 ± 0.02 |
|  | ***A. glutinosa*** | **No treatment** | 4.35 ± 0.25 | 0.95 ± 0.02 |
|  |  | **Woodchips** | 4.45 ± 0.16 | 0.96 ± 0.01 |
| **Unplanted control (t=0)** | | | 4.42 ± 0.07 | 0.97 ± 0.01 |
| **Unplanted control (t=f)** | | | 4.39 ± 0.09 | 0.96 ± 0.01 |
